# An Insulin Receptor-Binding Multifunctional Protein from *Tamarindus indica* L. Presents a Hypoglycemic Effect in a Diet-Induced Type 2 Diabetes—Preclinical Study

**DOI:** 10.3390/foods11152207

**Published:** 2022-07-25

**Authors:** Izael Costa, Mayara Lima, Amanda Medeiros, Lucas Bezerra, Paula Santos, Alexandre Serquiz, Maíra Lima, Gerciane Oliveira, Elizeu Santos, Bruna Maciel, Norberto Monteiro, Ana Heloneida Morais

**Affiliations:** 1 Biochemistry and Molecular Biology Postgraduate Program, Biosciences Center, Federal University of Rio Grande do Norte, Natal 59075-000, Brazil; izaelsousa@hotmail.com (I.C.); mayara.lima@yahoo.com.br (M.L.); amanda-nut@hotmail.com (A.M.); elizeu.ufrn@gmail.com (E.S.); 2 Chemistry Postgraduate Program, Science Center, Federal University of Ceará, Fortaleza 60020-903, Brazil; lucas.lima@alu.ufc.br (L.B.); norbertokv@ufc.br (N.M.); 3 Federal Institute of Education, Science and Technology of Rio Grande do Norte, Macau 59500-000, Brazil; paula.santos@ifrn.edu.br; 4 Nutrition Course, University Center of Rio Grande do Norte, Natal 59014-545, Brazil; alexandreserquiz@gmail.com; 5 Veterinary Medicine Course, Potiguar University, Natal 59056-000, Brazil; mairalima4@hotmail.com; 6 Nutrition Postgraduate Program, Center for Health Sciences, Federal University of Rio Grande do Norte, Natal 59075-000, Brazil; gercianesilva2@hotmail.com (G.O.); brunalimamaciel@gmail.com (B.M.); 7 Biochemistry Department, Biosciences Center, Federal University of Rio Grande, Natal 59075-000, Brazil; 8 Tropical Medicine Institute, Federal University of Rio Grande do Norte, Natal 59075-000, Brazil; 9 Nutrition Department, Center for Health Sciences, Federal University of Rio Grande do Norte, Natal 59075-000, Brazil; 10 Analytical Chemistry and Physical Chemistry Department, Science Center, Federal University of Ceará, Fortaleza 60020-903, Brazil

**Keywords:** trypsin inhibitor, tamarind, hypoglycemic agent, plant proteins, molecular docking simulation

## Abstract

The objectives of this study were to evaluate the hypoglycemic effect of the trypsin inhibitor isolated from tamarind seeds (TTI) in an experimental model of T2DM and the in silico interaction between the conformational models of TTI 56/287 and the insulin receptor (IR). After inducing T2DM, 15 male Wistar rats were randomly allocated in three groups (*n* = 5): 1—T2DM group without treatment; 2—T2DM group treated with adequate diet; and 3—T2DM treated with TTI (25 mg/kg), for 10 days. Insulinemia and fasting glucose were analyzed, and the HOMA-IR and HOMA-β were calculated. The group of animals treated with TTI presented both lower fasting glucose concentrations (*p* = 0.0031) and lower HOMA-IR indexes (*p* = 0.0432), along with higher HOMA-β indexes (*p* = 0.0052), than the animals in the other groups. The in silico analyses showed that there was an interaction between TTIp 56/287 and IR with interaction potential energy (IPE) of −1591.54 kJ mol^−1^ (±234.90), being lower than that presented by insulin and IR: −894.98 kJ mol^−1^ (±32.16). In addition, the presence of amino acids, type of binding and place of interaction other than insulin were identified. This study revealed the hypoglycemic effect of a bioactive molecule of protein origin from Tamarind seeds in a preclinical model of T2DM. Furthermore, the in silico analysis allowed the prediction of its binding in the IR, raising a new perspective for explaining TTI’s action on the glycemic response.

## 1. Introduction

Diabetes *mellitus* (DM) is characterized by a set of metabolic alterations that trigger increased serum glucose concentrations, causing hyperglycemia. This condition is caused by defects in insulin secretion and/or insulin resistance [1]. Among the different types of DM, there are a higher incidence and a higher prevalence of individuals diagnosed with type 2 diabetes mellitus (T2DM), a critical reason for morbidity and mortality [2].

Considering the risk factors that contribute to the emergence of T2DM, there are unhealthy lifestyle factors, such as a sedentary lifestyle and inadequate diet, especially related to the high consumption of foods with high glycemic indexes or high glycemic loads. Additionally, obesity triggers chronic inflammatory processes due to inflammatory cytokines, especially tumor necrosis factor-α (TNF-α), which induces the insulin resistance process [1,3].

Insulin is a hormone with 51 amino acid residues, which, when binding to its membrane receptor in specific cells, induces the activation of specific metabolic pathways to maintain glucose homeostasis [4,5,6]. The insulin receptor (IR) is formed by tetrameric proteins, consisting of two extracellular α subunits and two transmembrane β subunits, joined by disulfide bonds. The crystal structure of the extracellular domain of the IRΔβ (comprising domains L1, CR, L2, FnIII-1, -2, -3) showed that in the absence of insulin, the IRΔβ has an “inverted V” conformation [7]. When insulin binds to the α subunits of the IR, there is a conformational activation by induction, leading to changes in the activity of kinases in the β subunits, which undergo a process of self-phosphorylation in their tyrosine residues, afterward recruiting IR substrates (IRS). Those substrates are members of the IRS protein family, ranging IRS-1–6, which act as signals to organize and mediate specific signaling complexes [7,8].

With the knowledge related to the mechanism of action of insulin for glycemic control, this pathway and its respective proteins are excellent candidates for evaluating potential therapeutic agents and their possible mechanisms of action. In this sense, bioactive compounds and peptides of the most diverse origins presented hypoglycemic potential, and it was possible to identify that several of them that mimick insulin, binding to IR in a target cell [6,9,10,11].

The IR had its three-dimensional structure experimentally revealed, making it possible to estimate interactions and perform analyses on metabolic responses mediated by molecules that could interact with and activate it. In addition, the identification and evaluation of the effect of agonist molecules on the IR have gained prominence through computational simulations, using various analyses, in silico [12]. Examples of this are computer simulation studies that used the complex insulin structure at site 1 (L1) of terminal C of the extracellular α subunit (peptide αCT) of human IR (PDB ID 4OGA), allowing analyses that observed the relationships of other molecules with this region of human IR [13].

Among these molecules of protein origin that are present in plants and have a potential hypoglycemic effect, there is the tamarind seed trypsin inhibitor in its isolated, partially purified (TTI) [14] or purified (TTIp) from [15]. Carvalho et al. [14,15] studied Wistar rats with obesity and metabolic syndrome (MS) consuming a high-glycemic-index and high-glycemic-load diet (HGLI). They observed a lower mean fasting glucose concentration in animals treated with TTI and TTIp than in the control group, which did not receive treatment. However, the effects of this inhibitor on the release and action of insulin were not studied.

Another study with the TTI, nanoencapsulated in chitosan, and isolated whey proteins, showed that Wistar rats fed the HGLI diet exhibited a significantly lower fasting plasma glucose concentration, without changing the insulin concentration compared to the group of untreated Wistar rats, which also consumed the same HGLI diet [16,17].

The purified TTI (TTIp) was recently characterized, sequenced and had its molecular arrangement and conformation estimated [18,19]. Sequencing the molecule and its modeled structural conformation is safe, and can evaluate its pharmacological potential [15,18,19,20] as a ligand in the active site (pocket) of another molecule (receptor), hand as great relevance for a better understanding of its action on specific metabolic pathways, including those mediated by insulin. In silico studies have been considered a valuable tool with which to determine the potential of a particular biological activity and provide information about the possible interactions exhibited by the most diverse peptides [21,22,23,24]. Thus, testing the modeled structure of TTIp as a ligand of another molecule has great relevance for understanding its action on specific metabolic pathways, including those mediated by insulin.

In this study, TTI was analyzed as a hypoglycemic agent candidate. A preclinical study was conducted to evaluate the effects of TTI on fasting glucose, insulinemia, insulin resistance indexes, pancreatic β cell functionality and insulin sensitivity using a diet-induced T2DM model. In addition, a computational study of molecular docking and molecular dynamics simulation was performed, with TTIp model number 56 and conformation number 287 (TTIp 56/287) to evaluate the interaction of this protein with the IR.

## 2. Materials and Methods

### 2.1. Isolation of the Trypsin Inhibitor from Tamarind Seed (TTI)

For the experimental study, the TTI was obtained from tamarind fruit’s (*Tamarindus indica* L.) seeds, purchased from a local store, botanically identified by the Brazilian Institute of The Environment and Renewable Natural Resources (IBAMA) Natal/RN (Brazil). In this study, all the plant experiments complied with relevant guidelines and legislation in the National System of Management of Genetic Heritage and Associated Traditional Knowledge (SisGen), under the number AF6CE9C. All reagents used were analytical and obtained from SIGMA (St. Louis, MO, USA) and VETEC Química Fina Ltd. a© (Rio de Janeiro, Brazil).

The TTI was isolated from the cotyledons of tamarind seeds (*Tamarindus indica* L.), according to Carvalho et al. [14] TTI was obtained by submitting protein fraction 2 (F2: saturation range with ammonium sulfate from 30–60%) to affinity chromatography on trypsin inhibitor sepharose 4B; the chromatographic column was calibrated with 50 mM of Tris-HCl buffer, pH 7.5. The elution of the column adsorbed proteins occurred with HCl at 5 mM in a flow of 0.5 mL/min. For the evaluation of the inhibition activity against trypsin, bovine trypsin with N-benzoyl-DL-arginine-p-nitroanide (BApNA) at 1.25 mM was used as a substrate. This reaction was read in a spectrophotometer at 405 nm [25]. In the TTI isolation process, proteins were quantified using the Bradford method, using serum bovine albumin [26]. The degree of purity and molecular mass of TTI, with 15 μg/mL, were estimated by electrophoresis in non-reducing and non-reducing discontinuous polyacrylamide gel (SDS-PAGE), according to Laemmli [27], and silver stained according to Oakley, Kirsch, Morris [28].

### 2.2. Experimental Model

In this experiment, adult male rats (with 31 weeks and weighing on average 400 g) of the species *Rattus norvegicus* Wistar strain were used. T2DM was experimentally induced using the diet of high glycemic index and high glycemic load (HGLI) for 17 weeks, according to Carvalho et al. [14]. T2DM was classified according to Wang et al. [29]. The animals were kept in standard conditions of luminosity (12 h light/12 h dark) and temperature (23–25 °C). Water and food were offered ad libitum.

All animal experiments were performed in accordance with the relevant guidelines and regulations. The entire experiment was performed in accordance with the *ARRIVE* guidelines (*Animal Research: Reporting of* In Vivo *Experiments*) [30], and after approval by the Ethics *Committee* on the Use of Animals of Potiguar University (CEUA-UNP), under protocol number 019/2017.

After 17 weeks of the consumption of the HGLI diet for T2DM induction, veterinarians anesthetized the animals with 250 mg of tiletamine hydrochloride and 250 mg zolazepam hydrochloride, and after the onset of anesthesia, collected 1 mL of blood by cardiac puncture, stored in a specific collector tube. Then, the serum was separated by centrifugation at 30,100× *g* for 10 min and used to determine glucose concentration with *glucose oxidase Assay Kit* (Abnova-KA3945). The animals with fasting glycemia above 7.5 mmol/L, according to Wang et al. [29], were classified with T2DM and continued the experiment for 10 days as follows.

The animals classified as T2DM were randomly divided into three groups of diabetic animals and allocated into individual cages (19 cm × 12.5 cm × 7.5 cm). After an adaptation period of 5 (five) days, the experiment was conducted for 10 (ten) days, where daily, in the afternoon shift, TTI was administered by oral gavage (25 mg/kg), and the LABINA and HGLI diets were offered *ad libitum*, as established by Carvalho et al. [14], according to the following groups:

1. Untreated T2DM (*n* = 5): Received the HGLI diet + 1 mL of distilled water by gavage. This group was considered the diabetic, without treatment group.

2. T2DM treated with standard diet (*n* = 5): Received the LABINA diet + 1 mL of water per gavage. This group was considered the diabetic, treated with nutritionally adequate diet group.

3. T2DM treated with TTI (*n* = 5): Received the HGLI diet + 1 mL of TTI (25 mg/kg) per gavage. This group was considered the diabetic, treated with TTI group.

On the 11th day, at the end of the treatments, the animals were kept fasting for a period of 8 h. Then, veterinary physicians, blinded to the groups, anesthetized the animals with 250 mg of tiletamine hydrochloride and 250 mg of zolazepam hydrochloride. The blood was collected by cardiac puncture and stored in a specific collector tube; then, the serum was separated by centrifugation at 3000 rpm for 10 min and used to determine glucose and insulin concentration.

Fasting glucose was determined by the enzymatic colorimetric method of oxidase-peroxidase, using *glucose oxidase Assay Kit*: KA3945 (Abnova, Taipei, Taiwan), and insulin was quantified using the Rat/Mouse Insulin ELISA Kit: EZRMI-13K (Millipore^®^ Temecula, CA, USA).

To measure insulin resistance and pancreatic β functionality, the *Homeostasis model assessment for insulin resistance* (HOMA-IR) and the *Homeostasis model assessment β-cell function* (HOMA-β) [31] were calculated, using the following formulas:

HOMA-IR: fasting insulin (μUI/mL) × fasting glucose (mmol/L)/22.5

HOMA-β: (20 × Insulin μUI/mL)/(Glucose mmol/L − 3.5)

### 2.3. Molecular Docking and Molecular Dynamics Simulations

#### 2.3.1. Sourc of the Studied Molecules

In this study, we used the three-dimensional structure of TTIp, model number 56, and conformation number 287 (TTIp 56/287), according to Medeiros et al. [19]. The three-dimensional structure of the insulin receptor (IR) with insulin with a resolution of 3.50 Å used in this study was obtained from the RCSB Protein Database [32], with the code PDB ID: 4OGA, according to Menting et al. [13]. The IR structure (PDB ID 4OGA) used for molecular docking with TTIp 56/287 was prepared by removing the molecules of water, ions and other ligands present in the original structure, including insulin, described in the structure of Menting et al. [13]. In this structure, the L1 region of terminal C of the subunit α extracellular (peptide αCT) [13] is part of the IR isoform B [12,33].

#### 2.3.2. Molecular Docking between TTIp 56/287 and IR

The molecular docking study between the TTIp 56/287 molecule and IR (PDB ID 4OGA) (TTIp 56/287-RI) was performed using the online server Molecular Docking Algorithm Based on Shape Complementarity Principles (Patchdock) [34]. Subsequently, the TTIp 56/287-RI was refined using the Online Server Fast Interaction Refinement in Molecular Docking [35] docking solutions of protein–protein complexes. The TTIp 56/287-RI with the lowest energy (Supplement Table S2) was used as input for molecular dynamics simulations.

Molecular docking between insulin structure and the IR (Ins-IR) was not necessary because the original crystallographic structure of the IR was already interacting with insulin as described by Menting et al. [13].

#### 2.3.3. Molecular Dynamics Simulations of TTIp 56/287-IR (PDB ID:4OGA) and Ins-IR

Molecular dynamics simulations were performed using the *GROningen Machine for Chemical Simulations* (GROMACS) version 2018.4 [36], implemented with the AMBER03 [37] force field. Water molecules via the transferable intramolecular potential with 3 points (TIP3P) were used to solvate the simulated systems [38]. The neutralization of the systems was obtained by adding Na^+^ counterions. The leapfrog algorithm [39] was applied to integrate the motion equation motion with a time interval of 2.0 fs. Long-range interactions were modeled using particle-mesh Ewald sum (PME) [40] with a cut-off of 1.2 nm. The van der Waals interactions were also calculated using the same threshold. Bonds involving hydrogen atoms were constrained using the LINCS algorithm [41]. The Nosé–Hoover thermostat [42] was used to fix the system temperature of 310 K (36.85 °C), in all production simulations; and the system pressure in the simulations with a certain number of particles, constant pressure and temperature (NPT) was controlled using the Parrinello–Rahman barostat, using atmospheric pressure [43]. The geometry of the system was minimized by steepest descent algorithm for 10,000 steps with a tolerance of 10 kJ mol^−1^ nm^−1^, followed by the conjugated gradient algorithm [44] for 10,000 steps with a tolerance of 100 kJ mol^−1^ nm^−1^. Two short 100,000 ps equilibrium simulations with NVT and NPT ensembles were performed. Finally, a 150 ns production MD simulation using the NVT ensemble was performed in triplicate (TTIp 56/287-IR and Ins-IR), in order to determine the energies of interaction between the molecules and IR (PDB ID: 4OGA).

For equilibrium analysis over time, the van Hove correlation function, $G_{s}\left(\vec{r},t\right)$ was determined, with which the probability density of a particle *i* can be verified to travel a distance *r* in a time interval *t*, is defined as Equation (1) [45]:

(1) $$G_{s}\left(\vec{r},t\right)=\langle \frac{1}{N}\overset{N}{\underset{i=1}{\sum }}\delta \left(\vec{r}-\left|{\vec{r}}_{i}\left(t\right)-{\vec{r}}_{i}\left(0\right)\right|\right)\rangle \;$$

where ${\vec{r}}_{i}$ is the position of the particle *i* at time *t*, and δ is Dirac’s delta function. We used molecular dynamics trajectories recorded for up to 25 ns with a time step of 0.002 ps.

The total energy of interaction between the TTIp 56/287-RI and Ins-RI was analyzed using the interaction potential energy (IPE) (kcal mol^−1^) [Equation (2)] [46].

(2) $$IPE_{i,j}=\overset{N_{i}}{\underset{i}{\sum }}\overset{N_{j}}{\underset{i\neq j}{\sum }}V_{vdW}\left(r_{ij}\right)+V_{elec}\left(r_{ij}\right)\;\;\;\;$$

where *IPE_i,j_* is the interaction energy between a group of atoms (*i*) and a group of atoms (*j*); *N_i_* and *N_j_* are the total numbers of atoms in groups *i* and *j*; V*_elec_* and V*_vdW_* are the corresponding terms for the electrostatic contributions and the forces of van der Waals.

Protein–protein interaction figures were constructed using visual molecular dynamics (VMD) software [47]. Graphic representations were generated by GRaphing, Advanced Computation and Exploration of data (Grace) [48].

### 2.4. Statistical Analysis

The sample size was calculated considering a simple and random sampling (Cochran’s model) [49], and according to the principle of the 3Rs (substitution, reduction and refinement). The result was an “*n*” of 4.36 animals. For this, a physiologically significant difference was assumed in the parameters investigated, when the treatment (TTI) exerted a biological effect of 25% or more in relation to the group that did not receive TTI. The coefficient of early variation of 10% was adopted, with probability of error below 5% and a power of 90%.

The Shapiro–Wilk test was used to assess the normality of the data. As these data did not follow the normal distribution, nonparametric tests were performed for all variables. These were analyzed by means of the *Kruskal–Wallis* test, followed by the *Dunns’ post test*. Graph Pad Prism, version 5.0 (Graph Pad Software, San Diego, CA, USA), was used for data analysis. For all tests, *p* values below 0.05 were considered significant.

## 3. Results

### 3.1. Obtaining the Trypsin Inhibitor from Tamarind Seeds (TTI)

**Figure 1:**
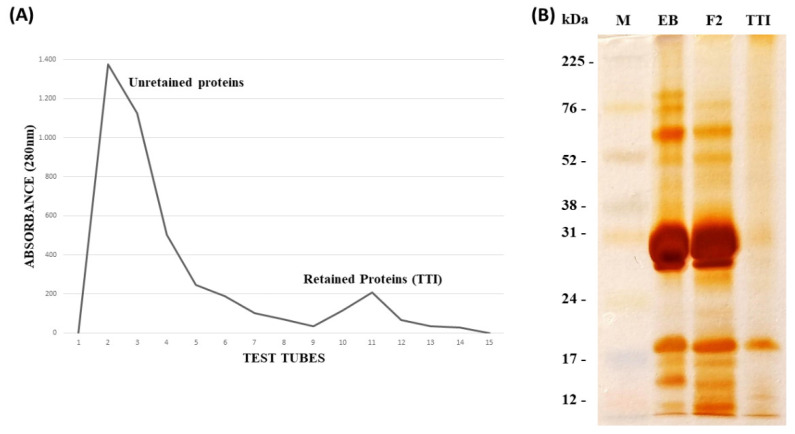
(**A**) Chromatographic profile of protein fraction 2 obtained from tamarind seeds (*Tamarindus indica* L.), which was submitted to affinity chromatography on trypsin inhibitor-sepharose 4B, calibrated with Tris-HCl buffer, at 50 mM, pH 7.5. The elution was performed using a 5 mM HCl solution, at a flow of 0.5 mL/me. (**B**) Denaturing polyacrylamide gel electrophoresis (SDS-PAGE at 12.5%, stained with silver nitrate). M: marker (RAINBOW); EB: crude extract (15 μg/mL); F2: protein fraction 2 (saturation with 30–60% ammonium sulfate) (15 μg/mL); TTI: trypsin inhibitor isolated from tamarind seeds (15 μg/mL).

The TTI was isolated from the cotyledons of tamarind seeds, after following the steps of protein extraction, fractionation with ammonium sulfate and affinity chromatography on trypsin inhibitor sepharose 4B (Figure 1A). The TTI isolation was visualized by SDS-PAGE at 12.5% (Figure 1B), and it was possible to identify a predominant protein at approximately 19 kDa.

### 3.2. Confirmation of T2DM in Wistar Rats after a 17-Week Period Consuming HGLI Diet

The fasting glucose of Wistar rats was high after the 17-week period with HGLI diet intake. All animals presented fasting glucose above 7.5 mmol/L (Table 1), a cutoff value for T2DM, according to Wang et al. [29].

### 3.3. Evaluation of Fasting Glucose, Fasting Insulinemia and HOMA-IR and HOMA-β Related to the Action and Functionality of Insulin in Wistar Rats with T2DM

**Figure 2:**
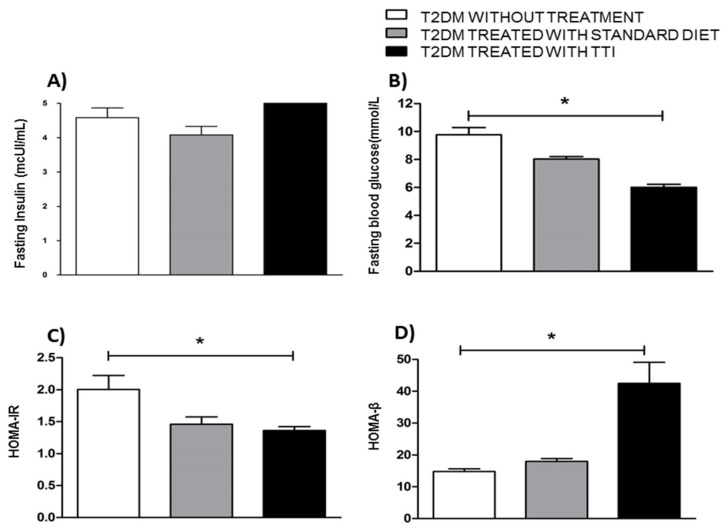
Fasting glucose and insulin concentrations, HOMA-IR and HOMA-β. (**A**) Fasting insulin (mcUI/mL). (**B**) Fasting blood glucose (mmol/mL). (**C**) Homeostasis model assessment for insulin resistance (HOMA-IR). (**D**) Homeostasis model assessment β-cell function (HOMA-β). The values are expressed as means ± standard deviations. Blood collection was performed on the eleventh day, after 8 h of fasting and 10 days of the experiment. Experimental groups: T2DM without treatment (*n* = 5): received HGLI diet + 1 mL of distilled water per gavage; T2DM treated with standard diet (*n* = 5): received LABINA diet + 1 mL of distilled water per gavage; T2DM treated with TTI (*n* = 5): received HGLI diet + 1 mL TTI (25 mg/kg) per gavage. The Shapiro–Wilk test was applied to evaluate the normality of the data. The data presented a nonparametric distribution, so the *Kruskal–Walls* test was used, followed by *Dunn’s* post-test, was used to detect significant differences between the studied groups. An asterisk indicates a *p* value * < 0.05 statistical difference found compared to the T2DM without treatment group. T2DM: type 2 diabetes *mellitus;* HGLI diet: high glycemic index diet and high glycemic load; LABINA diet (nutritionally adequate); TTI: trypsin inhibitor isolated from tamarind seeds.

The groups of animals with T2DM were evaluated after 10 days of experiment for: fasting insulinemia, fasting glycemia, HOMA-IR and HOMA-β. Regarding fasting insulinemia, there was no significant difference in insulin concentration between the group of animals treated with TTI in relation to the others (Figure 2A). In relation to fasting glucose (Figure 2B), the group of Wistar rats treated with TTI presented significantly lower fasting glucose than those untreated for T2DM (*p* = 0.0031).

In addition, the HOMA-IR (Figure 2C) of the animals treated with TTI was significantly lower than untreated animals (*p* = 0.0432), and like that of the animals with T2DM, treated with standard diet. The HOMA-β (Figure 2D), was higher in the group treated with TTI when than the untreated group (*p* = 0.0052).

### 3.4. Molecular Docking Analysis and Molecular Dynamics Simulation between TTIp 56/287 Systems with IR and between Insulin with IR

The conformation presenting a global energy of −100.6 kJ mol^−1^ in the molecular docking analysis of the TTIp 56/287-RI was used for molecular dynamics simulations in triplicate (Supplement Table S1). In addition, the Ins-IR (PDB ID 4OGA) was also submitted to molecular dynamics simulation in triplicate.

**Figure 3:**
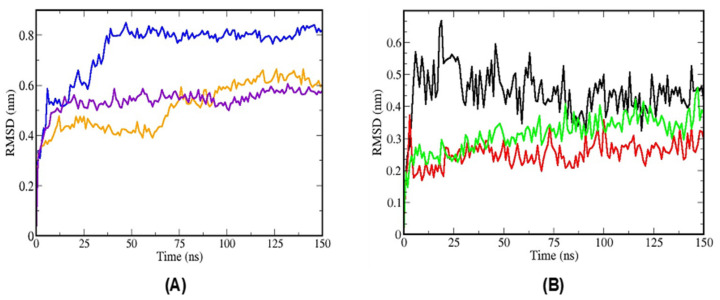
Root mean square deviations (RMSD) of molecular dynamics simulations. (**A**) RMSD of the molecular dynamics simulations of TTIp, model number 56, and conformation number 287 (TTIp 56/287) and insulin receptor (IR) (PDB ID 4OGA); (**B**) RMSD of the simulations of molecular dynamics of insulin (Ins) and the insulin receptor (IR) (PDB ID 4OGA), according to the structure described by Menting et al. [13]. Graphics generated by GRaphing, Advanced Computation and Exploration of data (Grace) in triplicate. Colors: lines in blue, orange and violet simulation of molecular dynamics in triplicate (TTIp 56/287-IR); lines in black, green and red simulation of molecular dynamics in triplicate (Ins-IR). TTIp: purified trypsin inhibitor of tamarind seed. ns: nanoseconds. nm: nanometers.

After the molecular dynamics simulations, which had as references the atoms of the TTIp 56/287 and insulin (Ins), it was possible to identify through the graphs of the potential energy of the root mean square deviation (RMSD) a structural equilibrium around 25 ns for the TTIp 56/287 (Figure 3A) and Ins (Figure 3B).

**Figure 4:**
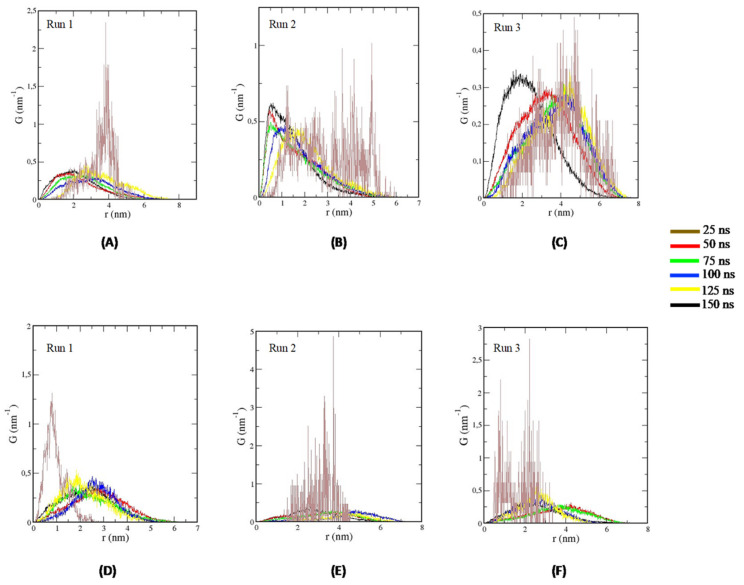
*Van Hove* correlations of molecular dynamics simulations at different times. (**A**–**C**) Correlations of *Van Hove* for TTIp model number 56, and conformation number 287 (TTIp 56/287) and insulin receptor (IR) (PDB ID 4OGA) at times: 25 ns (brown line); 50 ns (red line), 75 ns (green line), 100 ns (blue line), 125 ns (yellow line) and 150 ns (black line). (**D**–**F**): Correlations of insulin *van Hove* (Ins) and insulin receptor (IR) (PDB ID 4OGA) at times of: 25 ns (brown line), 50 ns (red line), 75 ns (green line), 100 ns (blue line), 125 ns (yellow line) and 150ns (black line). Graphics generated by GRaphing, Advanced Computation and Exploration of data (Grace) in triplicate. TTIp: purified trypsin inhibitor of tamarind seed. ns: nanoseconds. nm: nanometers.

Regarding the correlation of van Hove for TTIp 56/287 (Figure 4A–C) and insulin (Figure 4D–F), after 25 ns, there is a reduction in the dispersion in the systems composed of the TTIp 56/287-IR and Ins-IR, after initial t = 0.

**Figure 5:**
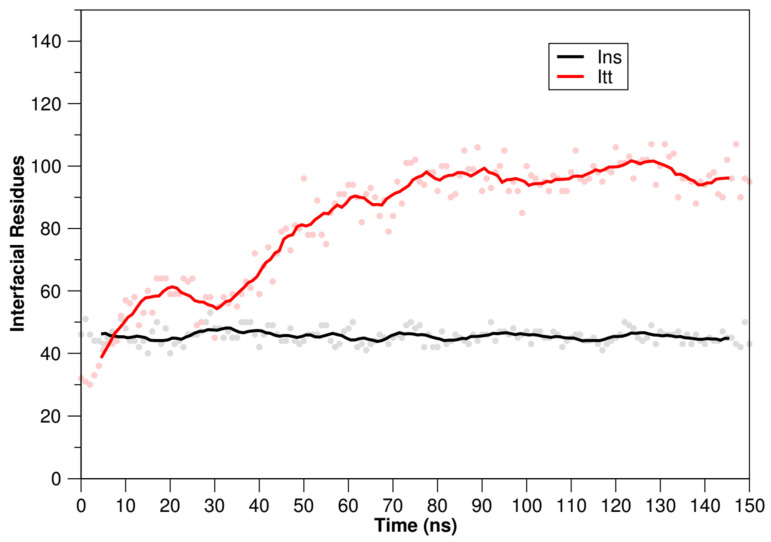
Interfacial residues of systems with TTIp 56/287-RI and Ins-IR after molecular dynamics simulations. Red line: interfacial residues of the system with the TTIp 56/287-IR: (TTIp model number 56, and conformation number 287 (TTIp 56/287) and the insulin receptor (IR) (PDB ID 4OGA)). Graphics generated by GRaphing, Advanced Computation and Exploration of data (Grace). Black line: interfacial residues of the system with the Ins-IR (insulin (Ins) and the insulin receptor (IR) (PDB ID 4OGA). TTIp: purified trypsin inhibitor of tamarind seed. ns: nanoseconds.

In addition, the number of interfacial residues was identified in the systems formed by the TTIp 56/287-IR and Ins-IR. The number of interfacial residues in the Ins-IR system remained unchanged throughout the molecular dynamics simulations (Figure 5, black line), whereas in the TTIp 56/287-IR system, the number of interfacial residues only reached equilibrium in about 80 ns (Figure 5, red line). Based on this, it was possible to infer that the time of simulation of molecular dynamics at 150 ns was enough to obtain a significant rearrangement and achieve structural equilibrium in the two simulated systems (Figure 5).

Based on the results of the RMSD graphs, van Hove distribution and interfacial residues of the systems with TTIp 56/287-IR and Ins-IR, calculations of the interaction potential energy (IPE) of the last 25 ns for both systems were performed.

The IPE calculated (standard deviation) between IR (PDB ID: 4OGA) and TTIp 56/287 was −1591.54 kJ mol^−1^ (±234.90), and that of insulin to IR was of −894.98 kJ mol^−1^ (±32.16). Thus, TTIp 56/287 exhibited greater affinity for IR compared to insulin, considering the calculated IPE.

After the analysis of the systems composed by the TTIp 56/287-IR and Ins-IR, the amino acid residues encompassed within a radius of 5.0 Å sphere with the lowest IPE values were identified (Supplement Table S1). The TTIp 56/287 encompassed 70 amino acid residues, being the residues of aspartate 7 (Asp7), glutamine 47 (Gln47), aspartate 145 (Asp145), arginine 59 (Arg59) and serine 34 (Ser34), the ones with the lowest IPE values. For insulin, which encompass 27 amino acid residues, asparagine 18 (Asn18), glutamate 17 (Glu17), cysteine 7 (Cys7), valine 26 (Val26) and leucine 31 (Leu31) had the lowest IPE values (Supplement Table S1).

**Figure 6:**
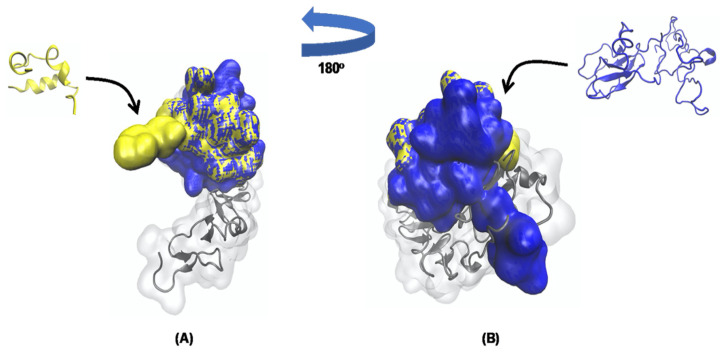
Geometric configurations of TTIp 56/28-RI and Ins-RI at 150 ns. (**A**) TTIp model number 56, and conformation number 287 (TTIp 56/287) and insulin receptor (IR) (PDB ID 4OGA). (**B**) Insulin (Ins) and insulin receptor (IR) (PDB ID 4OGA), according to the structure described by Menting et al. [13]. Images generated by visual molecular dynamics (VMD) software. Colors: gray: insulinreceptor (PDB ID 56/287); yellow: TTIp model number 56, and conformation number 287; blue: insulin (Ins). TTIp: purified trypsin inhibitor of tamarind seed. ns: nanoseconds.

After these analyses, it was possible to visualize the structural behavior and geometric configurations of the TTIp 56/287 and Ins-IR with 150 ns through the molecular dynamics simulations (Figure 6A,B), and to observe that TTIp 56/287 interacted with the IR in a region adjacent to insulin.

## 4. Discussion

T2DM is one of the main non-transmissible chronic diseases and presents hyperglycemia as its primary condition. This study revealed the hypoglycemic effect of a bioactive molecule of protein origin from Tamarind fruit seeds in a preclinical model of T2DM. Furthermore, the in silico analysis allowed the prediction of its binding to the IR, raising a new means to explain the TTI action on the glycemic response. These results are innovative and add to the comprehension of the bioactive effects of plant proteins in the most diverse diseases, among them is T2DM.

The animals in our study presented hyperglycemia above 7.5 mmol/L. This condition was induced by the chronic consumption of the HGLI diet, which is rich in simple and ultra-processed carbohydrates. The influence of the HGLI diet on the insulin resistance process, promoting cellular and metabolic alterations, has already been described by Luz et al. [50].

The HGLI diet caused hypertrophy of visceral adipocytes [50], a condition that might influence insulin activity. The emergence of insulin resistance was observed in the animals before and after treatment in the untreated group. Furthermore, excessive consumption of simple carbohydrates promotes the synthesis of free fatty acids, leading to increased plasma concentrations of inflammatory cytokines, such as tumor necrosis factor-α (TNF-α) and interleukin-6 (IL-6), among others [51]. Inflammatory cytokines activate the nuclear factor kappa B (NF-κB) pathway, leading to the phosphorylation of serine residues from the IRS, resulting in resistance to insulin action [52,53].

Nonetheless, the insulin concentrations presented in this study were not so high, and there were also no significant differences between the groups of animals submitted to different treatments. Nevertheless, TTI showed beneficial effects on insulin resistance and the functionality of pancreatic β cells through HOMA-IR and β indices, respectively, once they did not differ significantly compared to the animals submitted to conventional treatment (nutritionally appropriate diet). This reduction might be explained by the modulation of the inflammatory profiles of the animals mediated by TTI administration.

Previous studies using TTIp [20] and nanoencapsulated TTI [16] also presented beneficial effects of these molecules on pancreatic β cells of Wistar rats, fed for 17 weeks with the HGLI diet, the same one used to induce T2DM. The animals treated with nanoencapsulated TTI showed a better aspect regarding the pancreas and lesion of β cells, compared to animals that consumed the HGLI diet and were not treated with the inhibitor. Another study with TTI also showed the preservation of the structural characteristics of pancreatic cells in eutrophic Wistar rats [54]. Moreover, TTI and TTIp showed an anti-inflammatory effect in Wistar rats fed the HGLI diet, and showed lower concentrations of plasma TNF-α and the expression of its gene in visceral adipose tissue [14,15]. Thus, TTI may have promoted benefits to pancreatic β cells, possibly due to its anti-inflammatory potential, contributing to the hypoglycemic effect in animals treated with TTI.

Additionally, even animals treated with TTI presented higher HOMA-β indexes compared to the group of untreated animals. However, HOMA-β did not increase above 100, which would indicate dysfunction in β pancreatic cells [31]. Withal, after 17 weeks of the HGLI diet, fasting glucose concentrations were already altered when the animals were classified with T2DM.

Probably, there was impairment regarding the efficiency of insulin action in all groups with T2DM, corroborated by the observed impaired glycemic homeostasis. Further, another hypothesis is that TTI can probably modulate, along with insulin, the intracellular signaling pathway mediated by the insulin receptor activation because, in the in silico study, TTIp 56/287 was linked to the IR in a region adjacent to insulin. Therefore, performing binding affinity assays to investigate the direct interaction between TTI and IR will be necessary, and no less importantly, further in vivo study on TTI digestion by gastrointestinal tract enzymes will be needed. Furthermore, this direct in vivo evidence must be presented with analysis of glycated hemoglobin (HbA1c), the influence on lipid profile, glucose clamp study and glucose uptake by skeletal muscles or adipocytes, showing the effect of TTI on the IR signaling, these being future perspectives for studies with TTI.

In addition, for the first time, the TTI significantly promoted a lower plasma concentration of fasting glucose in animals treated with this molecule. The hypoglycemic potential of bioactive protein molecules of plant origin has been identified over the years; currently, many of them are active drugs for treating DM2 [6,55]. Moreover, the TTI partially purified, purified or nanoencapsulated, has been presented as a safe molecule, and no signs of toxicity were observed with the doses used in the present study for the same period [14,16,20,56,57].

These findings may corroborate those revealed in the study conducted by Xu et al. [58], in male Sprague–Dawley rats with Aloxan-induced T2DM and a diet rich in fat and simple carbohydrates after treatment with Ginseng oligopeptides (GOPs) for 52 weeks. These authors observed that GOP treatment corrected hyperglycemia in rats with T2DM, and that the HOMA-IR values were significantly lower, unlike HOMA-β, which was higher compared to the untreated diabetic group. Thus, the GOP protected β cells from deterioration, induced by alloxan, and thereby prevented insulin deficiency in these animals.

Some limitations of this study should be pointed out, such as the absence of a control group of eutrophic and healthy animals, to show more clearly the changes induced by the HGLI diet in the glycemic parameters of the animals. However, in a previous study [50] by the NutriSBioativoS research group, the biochemical parameters of eutrophic and healthy animals, living under the same conditions as this study, showed no changes, serving as a reference parameter of normality for this study.

According to Acquah et al. [59] and Costa et al. [6], bioactive molecules of protein origin, obtained from the most diverse origins, present great hypoglycemic potentials. In their reviews, these authors highlighted that they act in several ways to control hyperglycemia and diabetes: inhibition of digestive enzymes, glucagon-likepeptide receptor agonist 1 (GLP-1), inhibitor of dipeptidyl peptidase-IV and insulin-like peptides. Plus, they have potential for binding to the IR. All this justifies the interaction study by molecular dynamics simulations between TTIp 56/287 and IR.

Thus, as the structural conformation of TTIp was already known, along with the model that best adjusted to the parameters of molecular dynamics simulation (TTIp 56/287) interacting with trypsin [19], the knowledge of these characteristics allowed the bioprospection of a possible interaction between TTIp 56/287 and the IR (PDB ID 4OGA). In this study, using molecular docking, it was possible to observe the interaction between the two structural models, TTIp 56/287 and insulin, with the IR (PDB ID 4OGA), the latter being the same presented in the study by Menting et al. [13].

The IR has two isoforms, IR-A and IR-B, resulting from the alternative splicing of the IR gene. The only difference between these isoforms is the insertion of 12 amino acids (IR-B plus and IR-A minus 12 amino acids) in terminal C of the extracellular α subunit, called αCT peptide. This difference in sequence has a relatively subtle impact on ligand binding and intracellular signaling, but may, however, have important physiological consequences for the isoform involved in the insulin-mediated signal transduction process [12,33].

In the present study, the IR-B isoform of IR (PDB ID 4OGA) was used with the presence of αCT L1 domains, as described by Menting et al. [13], and this region is associated with the activation of secondary signaling pathways for GLUT-4 mediated glucose translocation in specific cells, such as liver and muscle cells.

Another vegetable that has been vastly studied in relation to its antidiabetic effect is the bitter melon (*Mamordica charantia* L.). In this plant, there are bioactive molecules with hypoglycemic potential; among them are the MC6 protein [60], the polypeptide-p [61] and the MC IR binding protein (mcIRBP) [62], in addition to the protein extract MCSE [63], which can be obtained in fruits and seeds. Some of these authors conducted in silico analysis with some of these proteins, with distinct molecular characteristics, to identify whether the hypoglycemic effect would be for its interaction with the IR.

In the study conducted by Lo et al. [63], the proteins that comprised the protein extract MCSE were identified, which presented hypoglycemic activity, and from this, these molecules were submitted to molecular docking analysis to identify their possible interactions with IR. For the analyses, the researchers used the PatchDock server to check the possible IT protein binding sites (PDBID 1VBW). This is one of the proteins of MCSE, which interacts with IR (PDBID 2DTG). They recognized greater interaction between IT protein and IR in the L1/CR region (N-terminal ri part formed by two domains with homologous leucines (L1 and L2), separated by a cysteine-rich (CR) region). In addition, they observed that ten IT amino acid residues interacted with eight residues located in the L1, CR and L2 domains of IR through hydrophobic interactions.

Lo et al. [62] analyzed the interaction between insulin receptor (IR)-binding protein (mcIRBP) and insulin with IR. They used the PatchDock server to predict the interaction sites between IR (PDBID 3LOH), mcIRBP protein (PDBID 1VBW) and insulin (PDBID 1JCO). They identified that mcIRBP interacted with the L1/CR/L2 segments of IR, and insulin with the α-CT segment of IR. In addition, these authors observed that the mcIRBP presented three distinct amino acid residue sequences (17–21, 34–40 and 59–66) that interacted with IR in the L1, CR and L2 regions, and the IR regions that were interacting with the mcIRBP were rich in leucines and cysteines, thereby being binding regions with hydrophobic nature. They also observed that mcIRBP interacted in different regions of insulin, in the IR, and activated the IR-mediated transduction signal with the activation of glucose and lipid metabolism genes, which, in turn, increased glucose uptake in cells.

In the present study, the potential of TTIp 56/287 as a ligand of the IR (PDB ID 4OGA) was revealed. Medeiros et al. [19], when performing the molecular dynamics simulations analysis between TTIp 56/287 and trypsin, identified that the IPE between these two molecules was −301.0128 kcal mol^−1^ and that the amino acid residues isoleucine (54), proline (57), arginine (59), arginine (63) and glutamine (78) from TTIp 56/287 had the strongest interactions with trypsin, especially the residues of arginine, an amino acid of a basic nature, so these authors infer that the active site of trypsin presented a negative character in the interaction with TTIp 56/287.

Just like in the study by Medeiros et al. [19], it was possible to verify that TTIp 56/287 also interacted with another molecule, and in this case, the receptor, IR (PDB ID 4OGA), and that amino acid residues that interacted with TTIp 56/287 and IR were different from those observed by Medeiros et al. [19]. However, in the present study, it was possible to notice that the amino acid residues that presented greater interactions with IR (PDB ID 4OGA) also showed electrostatic characteristics, and those identified by Medeiros et al. [19], reinforcing the multifunctional characteristic of TTI, already shown in preclinical studies, acting on inflammation and obesity [14,64]. Regarding insulin, in this study, amino acid residues with greater interaction with IR (PDB ID 4OGA) presented hydrophobic characteristics, corroborating what was identified by Lo et al. [62,63] in their studies with insulin mimetic proteins.

The crystalline structure of the extracellular domain of IR (isoform B) showed that the insulin molecule binds to a primary binding site, consisting of the L1 domain of the α-CT peptide, and this primary site is defined as IR site 1 [13]. Researchers, in different studies, have identified by single-particle electron microscopy that insulin binding to IR induces a major conformational change of the IR, modifying its conformation from “inverted V” shape to a “T” shape [64,65,66]. This conformational change of IR triggers a new interface of the insulin-IR, which will involve a handle of FnIII-1, a structure proposed to represent IR site 2, identifying that IR may be interacting with one or two insulin molecules, with each binding to a specific site, and activating the signaling cascade mediated by this hormone [64,66].

As in the studies cited, TTIp 56/287 interacted with the IR isoform B (specific IR region to promote insulin-mediated signal transduction) and insulin, but in a region adjacent to it. Thus, the data reinforced the hypothesis that insulin-mimetic molecules, when binding to a region other than that occupied by insulin in the IR, can also activate or modulate signaling pathways that promote glycemic control. As discussed earlier, proteins and bioactive peptides of protein origin bind to the insulin receptor, and in this study, TTIp 56/287, a structure of plant protein origin, was a ligand to the IR (PDB ID 4OGA) in a region adjacent to insulin.

Although TTIp 56/287 did not have a similar structure to insulin, it interacted with IR in a different region, and presented an insulin-mimetic action, shown through the preclinical analyses performed. Researchers state that insulin-mimetic plant proteins do not present essentially the same characteristics when compared to each other or to mammalian insulin, as observed in the present study [67,68,69]. However, they usually have one point in common: the ability to reduce blood glucose concentrations of laboratory animals with diabetes. In addition, these authors suggested that not all isolated proteins showed the presence of insulin-like antigenic epitopes. In contrast, they hypothesized plant proteins that are more structurally similar to the mammalian hormone and are recognized by anti-insulin antibodies will be also more likely to activate the insulin-mediated signaling cascade because these pathways are induced by the specific interaction of insulin with its membrane receptors [67,68].

This characteristic structural similarity present in plant proteins was already reported, such as in the purified hypoglycemic proteins of *V. unguiculata*, which contain identical amino acid sequences and molecular masses very similar to that of bovine insulin and are recognized by anti-insulin antibodies [70]. In addition to this, the proteins of the leaves of *C. Igneus* contain epitopes similar to this hormone [71]; and *conglutin-γ*, a glycoprotein obtained from lupin seeds, promoted increased glucose transport and glut-4 translocation by stimulating the same insulin signaling pathways binding to its membrane receptor [72].

Naturally, molecules that present insulin-like action are present in the body and bind in distinct regions of the IR at the same time as insulin, playing glycoregulatory functions. Bhaskar et al. [73] identified a human monoclonal antibody, XMetA, which presents insulin-like action, binding with high affinity to the IR, and triggers a glycoregulatory activity. This molecule did not have a similar conformational structure to insulin, but could bind to different regions of the RI at the same time as insulin. Thus, the behavior of the TTIp 56/287-Ins-RI should be deeply studied, defining the insulin-mimetic characteristic of TTI, thereby gaining greater knowledge about the behavior of TTI in T2DM.

## 5. Conclusions

The TTI presented great potential for stimulating glucose uptake in cells sensitive to insulin, as it bound in silico to the IR. Furthermore, the hypoglycemic effect and modulation of the glycemic response in the pre-clinical model, and the in silico analysis showing the interaction of the TTIp 56/287 with the IR (PDB ID 4OGA), support the possibility of TTI use in studies aiming the control of diabetes.

## Figures and Tables

**Table 1:** Fasting glucose concentrations of male adult Wistar rats submitted to T2DM induction with an HGLI diet after 17 weeks. Animals were in collective cages (545 mm × 395 mm × 200 mm), up to three animals per cage.

Rats	Fasting Glucose (mmol/L)
Rat 1	9.30
Rat 2	7.85
Rat 3	8.92
Rat 4	7.73
Rat 5	7.85
Rat 6	8.51
Rat 7	9.37
Rat 8	9.73
Rat 9	7.54
Rat 10	10.23
Rat 11	12.00
Rat 12	9.44
Rat 13	9.41
Rat 14	8.38
Rat 15	11.14

T2DM: diabetes *mellitus* type 2; mm: mm; mmol: milimol; L: liter; HGLI diet: diet with a high glycemic index and high glycemic load.

## Data Availability

The data presented in this study are available on request from the corresponding author.

## Supplementary Materials

The following supporting information can be downloaded at: https://www.mdpi.com/article/10.3390/foods11152207/s1, Figure S1: Full-length gels. Table S1: Interaction Potential Energy (IPE) of each amino acid residue between ITTp 56/287-IR and Ins-IR complexes as a function of the time of molecular dynamics simulation. Table S2: Structures generated from the TTIp 56/287-IR complex and their global energies generated by FireDock.
