# Supplementary material for: An Insulin Receptor-Binding Multifunctional Protein from Tamarindus indica L. Presents a Hypoglycemic Effect in a Diet-Induced Type 2 Diabetes—Preclinical Study
Source: Foods. 2022 Jul 25;11(15):2207. doi: 10.3390/foods11152207 (PMC9332146; doi:10.3390/foods11152207)
Supplement: Supplementary file 1 [file foods-11-02207-s001.zip › foods-1784211-supplementary.pdf]

Supplementary Materials:

Figure S1. Full-length gels.

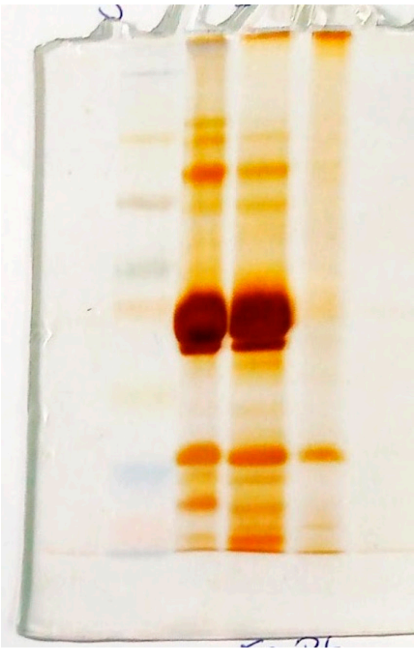

Table S1. Interaction Potential Energy (IPE) of each amino acid residue between ITTp 56/287-IR and Ins-IR complexes as a function of the time of molecular dynamics simulation.

ITTp 56/287		Insulin	
**Waste**	**EPI / (kJ mol-1)**	**Waste**	**EPI / (kJ mol-1)**
Lys154	0.66 (1.10)	Gln15	-0.77 (0.58)
Ile54	-0.67 (2.16)	Tyr14	-0.88 (1.09)
Gly32	-0.69 (1.27)	His24	-1.26 (13.67)
Trp181	-0.74 (0.88)	Leu16	-1.56 (0.54)
Ile50	-0.85 (4.20)	Cys33	-2.18 (3.56)
Trp92	-0.88 (0.57)	Leu25	-7.03 (3.10)
Ile20	-1.15 (2.46)	Gly37	-7.40 (6.19)
Glu180	-1.20 (7.36)	Leu29	-8.53 (4.06)
Asp51	-1.28 (9.71)	Gly23	-8.85 (15.17)
Asn76	-1.31 (1.56)	Gly34	-10.22 (12.17)
Gln25	-1.40 (2.41)	Thr41	-10.26 (22.64)
Leu43	-1.47 (1.20)	Val3	-20.49 (12.48)
Pro89	-1.83 (0.84)	Ile2	-22.26 (7.18)
Thr44	1.86 (1.87)	Tyr30	-25.77 (9.73)
Pro49	-1.89 (1.60)	Ser9	-28.70 (40.45)
Tyr19	-2.71 (4.34)	Asn21	-34.44 (8.85)
Gln123	-2.84 (3.08)	Tyr19	-35.31 (5.95)
Cys85	-3.13 (5.22)	Phe38	-39.00 (16.91)
Gly29	-3.17 (4.60)	Cys20	-39.14 (3.80)
Ala84	-3.49 (1.96)	Tyr40	-42.40 (11.25)

Tyr18	-3.53 (11.27)	Glu27	-42.63 (42.87)
Ala23	-3.80 (4.99)	Glu35	-48.38 (47.15)
Pro22	-3.85 (4.24)	Phe39	-50.82 (7.53)
Ile183	-5.58 (11.11)	Asn18	-53.60 (11.06)
Ile69	-4.65 (2.58)	Glu17	-139.80 (26.28)
Gln24	-6.58 (8.26)	Cys7	-147.72 (46.89)
Ala116	-6.88 (1.86)	Val26	-577.36 (10.49)
Ala86	-9.16 (3.22)	Leu31	-579.75 (16.49)
Pro11	-9.56 (2.95)		
Glu153	-9.62 (5.31)		
Ala90	-10.51 (3.03)		
His4	-14.50 (4.65)		
Leu73	-14.63 (4.74)		
Thr3	-14.94 (4.87)		
Pro87	-15.13 (4.43)		
Val10	-15.26 (4.32)		
Leu31	-15.49 (5.79)		
Ile77	-15.88 (8.45)		
Ala82	-16.23 (6.59)		
Leu21	-16.46 (8.81)		
Pro83	-17.22 (6.40)		
Asp1	-18.83 (11.70)		
Val3	-21.15 (8.83)		
Gly8	-22.61 (14.36)		
Val45	-22.76 (7.18)		
Ile81	-23.24 (13.26)		
Val184	-23.64 (43.70)		
Thr80	-26.04 (22.59)		
Ala72	-26.36 (7.99)		
Thr2	-27.86 (15.64)		
Leu75	-28.91 (5.34)		
Lys27	-29.15 (43.25)		
Asp5	-29.70 (15.34)		
Asp111	-29.80 (28.32)		
Gln9	-30.64 (28.76)		
Leu121	-33.69 (15.36)		
Lys102	-34.96 (52.50)		
Thr48	-37.85 (22.73)		
Ala64	-40.83 (10.32)		
Glu162	-45.12 (47.35)		
Glu78	-46.97 (41.60)		
Thr70	-48.99 (17.43)		
Lys120	-59.70 (42.86)		

Asn151	-73.76 (10.16)		
Phe60	-75.43 (19.17)		
Asp7	-86.17 (39.96)		
Gln47	-91.16 (32.03)		
Asp145	-106.28 (12.26)		
Arg59	-275.61 (58.36)		
Ser34	-323.28 (46.58)		

Legend: Ala: Alanina; Asp: Asparagine; Cis or Cys: Cysteine; Asp: Aspartate (Aspartic acid); Glu: Glutamate (Glutamic Acid); Fen or Phe: Phenylalanine; Gli or Gly: Glycine; His: Histidine; Ile: Isoleucine; Lis or Lys: Lysine; Leu: Leucine; Met: Methionine; Asn: Asparagine; Pro: Proline; Gln: Glutamine (Glutamide); Arg: Arginine; Ser: Serina; Tre or Thr: Threoin; Val: Valina; Trp: Tryptophan; Tir or Tyr: Tyrosine; Glu: Glutamine or Glutamate. ITTp 56/287-IR: [ITTp model number 56, and conformation number 287 (ITTp 56/287) and insulin receptor (IR) (PDB ID 4OGA)]. Ins-RI: [insulin (Ins) and insulin receptor (IR) (PDB ID 4OGA]. ITTp: purified trypsin inhibitor of tamarind seed.

Table S2. Structures generated from the TTIp 56/287-IR complex and their global energies generated by *FireDock*.

**Classification**	**Solution number**	**Global Energy**
1	10	-24,09
2	8	-4,35
3	7	6,15
4	4	7,23
5	2	9,34
6	9	24,91
7	6	55,13
8	5	199,83
9	1	246,82
10	3	389,07

Legend: ITTp 56/287-IR: [ITTp model number 56, and conformation number 287 (ITTp 56/287) and insulin receptor (IR) (PDB ID 4OGA)]. ITTp: purified trypsin inhibitor of tamarind seed.
